# Human Umbilical Cord Blood Cells Restore Brain Damage Induced Changes in Rat Somatosensory Cortex

**DOI:** 10.1371/journal.pone.0020194

**Published:** 2011-06-01

**Authors:** Maren Geißler, Hubert R. Dinse, Sandra Neuhoff, Klaus Kreikemeier, Carola Meier

**Affiliations:** 1 Institut fur Neuroinformatik, Neural Plasticity Lab, Ruhr-University, Bochum, Germany; 2 Department of Neuroanatomy and Molecular Brain Research, Ruhr-University, Bochum, Germany; Dalhousie University, Canada

## Abstract

Intraperitoneal transplantation of human umbilical cord blood (hUCB) cells has been shown to reduce sensorimotor deficits after hypoxic ischemic brain injury in neonatal rats. However, the neuronal correlate of the functional recovery and how such a treatment enforces plastic remodelling at the level of neural processing remains elusive. Here we show by in-vivo recordings that hUCB cells have the capability of ameliorating the injury-related impairment of neural processing in primary somatosensory cortex. Intact cortical processing depends on a delicate balance of inhibitory and excitatory transmission, which is disturbed after injury. We found that the dimensions of cortical maps and receptive fields, which are significantly altered after injury, were largely restored. Additionally, the lesion induced hyperexcitability was no longer observed in hUCB treated animals as indicated by a paired-pulse behaviour resembling that observed in control animals. The beneficial effects on cortical processing were reflected in an almost complete recovery of sensorimotor behaviour. Our results demonstrate that hUCB cells reinstall the way central neurons process information by normalizing inhibitory and excitatory processes. We propose that the intermediate level of cortical processing will become relevant as a new stage to investigate efficacy and mechanisms of cell therapy in the treatment of brain injury.

## Introduction

Cell therapy has become a promising therapeutic option for many human diseases, injuries and stroke [1], [2], [3], [4], [5], [6], [7]. The intraperitoneal transplantation of the mononuclear fraction of human umbilical cord blood (hUCB) cells has been successfully applied in the treatment of perinatal hypoxic ischemic brain injury [8], [9], models of stroke [10], [11], [12] or other diseases [13], [14]. There is a discussion whether beneficial effects of transplantation are directly mediated through differentiation processes and/or due to release of multiple factors at the lesion site [3]. However, while the hUCB treatment ameliorates motor deficits and improves behavioural outcome, the physiological basis of functional restoration remains elusive.

Brain injuries lead to a dysbalance of excitation and inhibition [15], [16], [17], [18], [19], [20], due to a dramatic increase in excitation and a decrease in inhibition leading to hyperexcitability of the surrounding tissue of the damaged area. Besides shrinkage of cortical maps and enlargement of receptive fields (RFs) representing affected body parts [21], [22], [23], [24], reduced paired-pulse suppression has been described indicative of cortical excitability changes [18]. At a cortical level, spatio-temporal processing is based on local operations of excitatory and inhibitory interactions [25], [26], [27], [28], [29]. Behaviourally relevant descriptors of cortical processing are receptive fields and cortical maps. Many studies have shown that the size of cortical representations predicts perceptual and behavioural performance [30], [31], [32]. The somatosensory cortex is the main cortical recipient of cutaneous information from the skin. Therefore, understanding tactile processing, which governs tactile perception and sensorimotor behaviour, has centered on characterizing size and structure of cortical receptive fields (RFs). Paired-pulse-behaviour, which characterizes the suppression elicited by pairs of stimuli presented at short interstimulus intervals (ISI), is discussed with respect to temporal processing, and provides information about cortical excitability and cortical inhibitory processes [33], [34], [35]. We therefore hypothesized that beneficial effects of hUCB treatment, which become apparent behaviourally, must be observable at an intermediate processing level and should therefore be measurable as a normalization of cortical receptive fields, maps and temporal processing abilities. To study the cortical changes evoked by lesion and following hUCB-treatment we performed electrophysiological in vivo recordings in primary somatosensory cortex (SI). To analyze spatial and temporal tactile processing we measured in both hemispheres the size of the cortical hind paw representation, the size of receptive fields (RFs), and cortical excitability with a tactile paired-pulse stimulation protocol. Using a combination of immunohistochemical, electrophysiological and behavioural studies in a rat model of hypoxic ischemic brain injury, we here demonstrate that hUCB transplantation largely normalizes spatial and temporal cortical processing.

## Results

**Figure 1:**
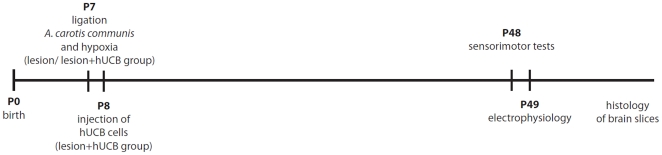
Time line of the experiments. Time line of the experiments, indicating the time points of the surgery, the treatment and the electrophysiological and behavioural experiments.

**Figure 2:**
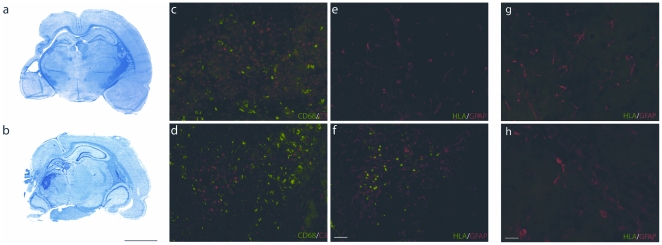
Histological and immunohistological analysis of brains. In Klüver-Barrera stained sections animals of the lesion group (a) and the transplantation group (b) showed clear cystic infarction within the left hemispheres, accompanied by an enlargement of the lateral ventricle and partial destruction of the hippocampus. Immunohistochemical detection of CD68 (green) and cleaved-caspase-3 expression (red) point to the occurrence of inflammation and apoptotic cell death in left hemispheres of animals of the lesion group (c) and transplantation group (d). In contrast, left hemispheres of control animals and right hemispheres of all animals were devoid of CD68 and cleaved-caspase-3 immunosignals (not shown). Immunohistochemical detection of HLA-DR demonstrated the presence of hUCB cells in the vicinity of the lesion in the transplantation group only (f), which are absent in the lesioned brain (e). No HLA-DR positive cells could be detected contralateral to the lesion in both the groups of lesioned (g) and transplanted rats (h). Scale bar left: 5 mm, scale bar right: 50 µm.

Intraperitoneally transplanted hUCB cells migrated to the lesion and were detected in the vicinity of the insult in all animals of the transplantation group (for a time line of the experiments see Fig. 1). This was confirmed by immunohistochemical staining with antibodies recognizing the human leukocyte antigen (HLA)-DR (Fig. 2e,f). In the contralateral hemisphere, no migrated cells could be detected (Fig. 2g,h). Furthermore, histological analysis of lesioned and control brains was performed to assess the extent and location of the insult. In Klüver-Barrera stained sections of the lesion and transplantation group, the lesion was clearly detectable, of identical volume (lesion group: 114.14±11.7 mm^3^; transplantation group: 110.68±11.87 mm^3^) and comprised cortex, hippocampus, and basal ganglia of left hemispheres (Fig.2). All control animals had morphologically intact brains. The insult was further assessed immunohistochemically, demonstrating the presence of activated microglia using anti-CD68 antibodies, and the occurrence of apoptotic cells death, assessed by detection of cleaved-caspase-3.

**Figure 3:**
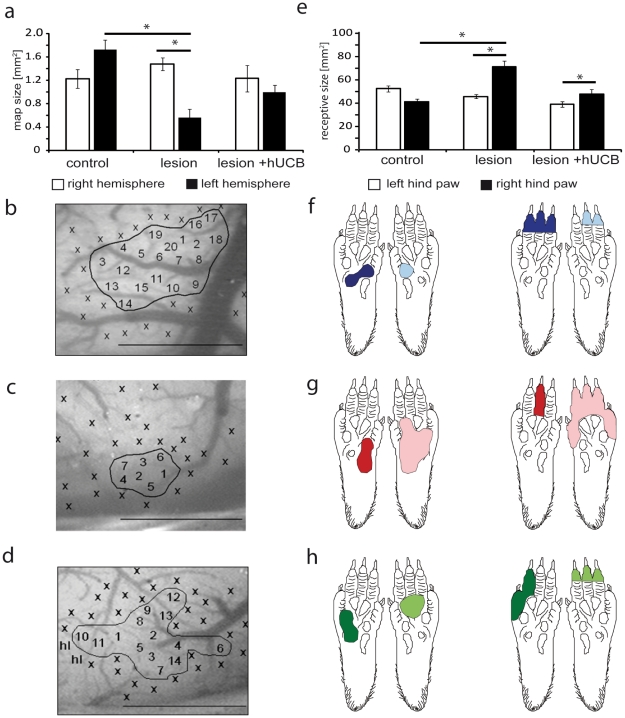
Effects of hypoxic ischemic brain injury and hUCB treatment on receptive field (RF) and cortical map size. a) In lesioned rats the size of the left cortical hindpaw (HP) representation was significantly reduced after hypoxic ischemic brain injury (HI) (p = 0.005 vs. controls, p = 0.004 vs. contralateral hemisphere). Treatment with hUCB cells prevented map changes in the left cortical HP representation. b)–d) images of the cortical surface of the left hemisphere of a control (b), lesioned (c) and hUCB treated rat (d). Numbers indicate penetration sites, x indicate non-cutaneous responses, hl hindlimb. Borders of the maps are outlined. Scale bar 1 mm. e) In lesioned rats RF size of the right HP was increased (p = 0.007 vs. controls, p = 0.03 vs. HP ipsilateral to the lesion). hUCB treatment lead to moderate RF increase, not significantly different from controls (p = 0.558). Bars represent s.e.m. f–h) examples of RFs on the right and left HP for a control (f), lesioned (g) and hUCB treated rat (h). Number of rats used: control group n =  10, lesion group n =  17, hUCB group n =  6. A total of 975 RFs were recorded (left hemisphere: control 127: lesioned 97, treated 85; right hemisphere: control 192, lesioned 327, treated 147).

In lesioned animals, hypoxic ischemic brain damage to the left hemisphere resulted in a significant reorganization of the lesioned side of the brain, indicated by a decrease of the size of the cortical hindpaw representation (Fig. 3a and b). Electrophysiological mapping revealed that size of the left cortical hind paw representation (0.56±0.14 mm^2^) was significantly smaller as compared to control rats (1.73±0.17 mm^2^) (p =  0.005), while right hemispheres were not affected by the lesion (control animals: 1.23±0.16 mm^2^; lesion animals: 1.5±0.11 mm^2^, Fig. 3). In contrast, in left hemispheres of hUCB treated animals, despite the apparent brain damage the average map size of 1.0±0.26 mm^2^ did not differ significantly from that observed in control animals (1.73±0.17 mm^2^). Accordingly, there were no side-to-side differences in hUCB treated animals (right hemisphere: 1.2±0.22 mm^2^, left hemisphere: 1.0±0.26 mm^2^, p =  0.366), indicating that the lesion-induced shrinkage of the map could be prevented or substantially ameliorated by the treatment.

### Receptive field size is rescued following hUCB injection

Additional beneficial effects of the treatment were detected by measuring receptive field (RF) size. Analysis of RFs revealed that hypoxic ischemic brain damage led to a significant increase in RF size in neurons recorded in the lesioned hemispheres (72±4.63 mm^2^) as compared to left hemispheres of controls (42±2.18 mm^2^). In lesioned animals, no changes of RF size were observed in neurons of the right, intact hemisphere representing the left hind paw (46±1.74 mm^2^). Treatment with hUCB cells led to the restoration of normal RF size (48±4.63 mm^2^) in the left hemisphere, which was not significantly different from controls (42±2.18 mm^2^).

**Figure 4:**
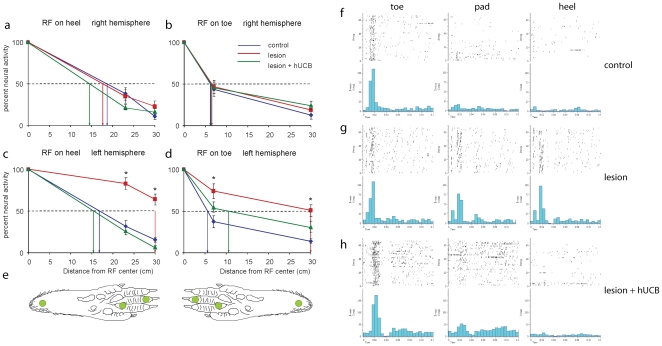
Length profiles of RFs. Percent activity evoked at three locations along the distal-proximal axis of the paw normalized to the activity recorded at the RF center. Data for RFs located on the heel are shown in a) and c), data for RFs with centers on the toe are depicted in b) and d). Top: recordings from the right, intact hemisphere, bottom: recordings from the left hemisphere. Stimulation locations on the paw are shown in the figurines in e). The distance from the RF centre at which 50% of neuronal activity (dashed line) was reached is indicated by arrows. Error bars represent s.e.m. For stimulation of the left paw in all groups comparable length profiles were obtained. In contrast, following stimulation of the right paw, significant differences (asterisks indicate significant differences p<0.05) were found for the length profiles obtained for control vs. lesioned animals, and for lesioned vs. treated animals, but not between control and treated rats. PSTHs recorded from neurons in the left hemisphere, whose RFswere located on the toes in control (f), lesioned (g) and transplanted rats (h) following tactile stimulation at the toe, pad, and heel. Whereas the activity evoked by stimulation on the pad and heel is low in controls (f), we found substantially enhanced activity after pad and heel stimulation in lesioned rats (g). In hUCB treated animals the activity pattern was comparable to control animals lacking the activation observed after pad and heel stimulation found in lesioned rats (h). Number of rats used: control group n =  7, lesion group n =  10, hUCB group n =  5. A total of 153 length profiles were recorded (left hemisphere: control 23: lesioned 24, treated 30; right hemisphere: control 24, lesioned 24, treated 28.

To scrutinize the RF-data obtained from handplotting, we additionally measured RF-length profiles using computer controlled stimulation based on recordings of PSTHs at 3 defined locations (toe, pad, heel, cf. Fig. 4 e) along the proximal-distal axis of the hindpaw. Examples of PSTHs are shown in Fig. 4f–h, which were recordedin the left hemisphere for neurons whose RF centres were located on the toes. In the lesioned animals, we recorded high levels of spiking activity following stimulation at the pad or heel indicative of large RF dimensions. In the hUCB treated animals, this abnormal response behaviour was no longer observed. As a measure of RF length we calculated that distance from the RF-center where neuronal responses in the PSTHs reached 50% of the maximal activity recorded from the RF-center (Fig. 4a–d). Average RF-length profiles obtained for the control and the treated animals were not different (p = 0.141 when RF centres were on the toes, and p = 0.830, when RF centres were on the heel), but significant differences were found between the control and the lesioned animals (p = 0.005 when RF centres were on the toes, and p<0.001when RF centres were on the heel) as well as between the lesioned and the treated animals (p<0.001for RF centres on the heel, and p<0.001for RF centers on the toes). These data confirm the observations obtained from handplotting that RF size in the treated animals do no longer show signs of lesion-induced RF-enlargement.

Cutaneous RFs in somatosensory cortex are contralaterally organized with no responsiveness of cortical neurons to ipsilateral sensory stimulation [36]. We therefore tested whether the lesion or the hUCB treatment resulted in the emergence of ipsilateral cortical representations, which, however, was not the case in any of the 333 recording sites tested for bilateral responses.

### Cortical temporal processing is normalized after HUCB transplantation

**Figure 5:**
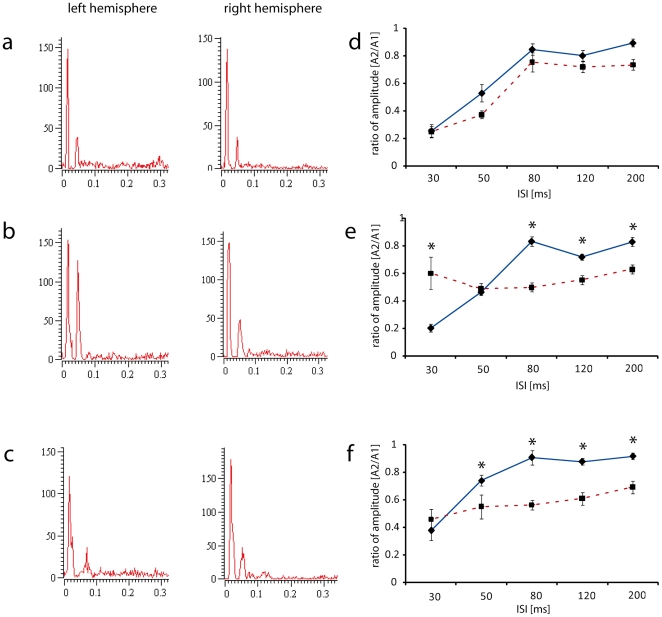
Effects of hypoxic ischemic brain injury (HI) and hUCB treatment on paired-pulse behaviour. a–c) PSTHs recorded in the left and right hemisphere of control (a), lesioned (b) and hUCB treated rats (c) at an ISI of 30 ms. Paired-pulse suppression is lost after HI, but restored after hUCB treatment. d–f) Averaged paired-pulse suppression (amplitude ratio - 2nd amplitude/1st amplitude) as a function of ISI for control (d), lesioned (e) and hUCB treated rats (f). Straight line: right, dashed line left hemisphere, asterisks indicate significant interhemispheric differences (p<0.05). The hyperexcitability observed at short ISIs in lesioned animals was ameliorated after hUCB treatment. Bars represent s.e.m. Number of rats used: control group n =  10, lesion group n =  17, hUCB group n =  6. A total of 386 paired pulse curves describing paired pulse behaviour at five different ISIs were recorded (left hemisphere: control 53: lesioned 51, treated 41; right hemisphere: control 65, lesioned 129, treated 47).

The paired pulse behaviour at short ISIs typically found in control animals consisted of a strong suppression of the second response. This behavior was quantified by calculating the ratio between the second and the first response amplitude (average ratio ISI_30_: 0.24±0.04, Fig. 5). At longer ISIs, suppression became increasingly smaller as indicated by increasingly higher ratios (ratio ISI_50_ 0.37±0.04, ratio ISI_80_ 0.75±0.07, ratio ISI_120_ 0.72±0.04, ratio ISI_200_ 0.73±0.04, Fig. 5).

In the left hemisphere of the lesioned animals, a qualitatively different paired-pulse-behaviour was observed. At short ISIs, the suppression of the second response was significantly reduced indicative of severe cortical hyperexcitability (ratio ISI_30_ 0.6±0.16) compared to the strong suppression found in the right hemisphere (ratio ISI_30_ 0.2±0.03; p = 0.047) or compared to the left hemispheres of controls (ratio ISI_30_ 0.24±0.04; p = 0.049). On the other hand, at longer ISIs, the responses recorded in the lesioned hemisphere showed even more suppression than typically observed in healthy controls, or in the contralateral right hemisphere (lesioned animals, left hemisphere: ratio ISI_50_ 0.5±0.06; ratio ISI_80_ 0.5±0.05; ratio ISI_120_ 0.6±0.05; ratio ISI_200_ 0.6±0.05). After the hUCB treatment, the reduced paired-pulse suppression observed at ISI_30_in lesioned animals was largely normalized. As a result, we no longer observed significant differences at an ISI of 30 ms between the right and the left, injured hemisphere (left hemisphere: ratio ISI_30_ 0.46±0.07, right hemisphere ratio ISI_30_ 0.38±0.07). The restoration of an almost normal paired pulse behaviour at ISI_30_was further supported by the lack of significant differences between treated animals (ratio ISI_30_ 0.46±0.07) and controls (ratio ISI_30_ 0.24±0.04; p =  0.108). In contrast, the paired-pulse suppression observed at ISIs 80, 120 and 200 remained lower as in control animals. The same observations were made when analyzing response integrals instead of amplitudes (see Methods).

### The beneficial effects of hUCB transplantation are clearly detectable in the sensorimotor outcome

**Figure 6:**
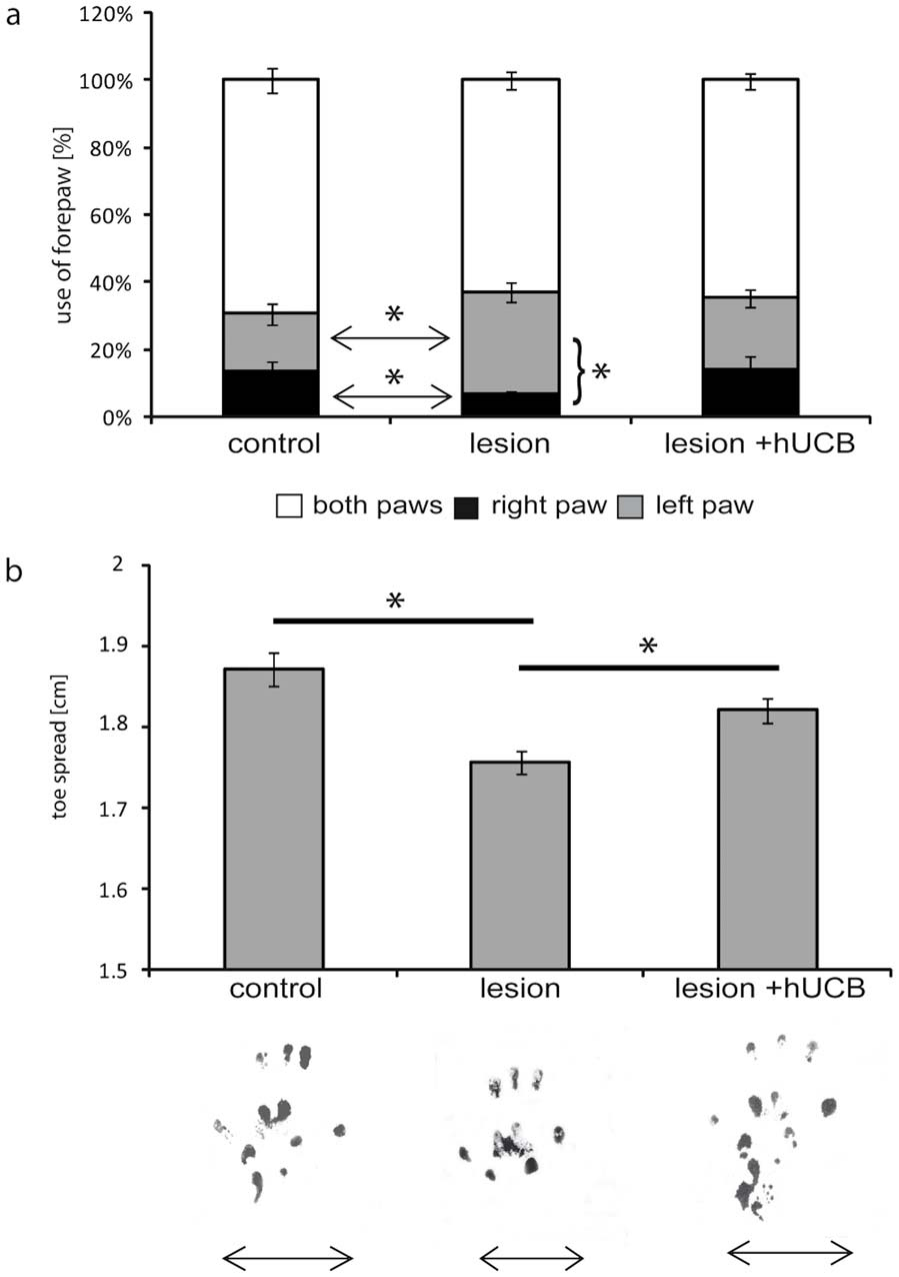
Assessment of the sensorimotor skills. The beneficial effects of hUCB treatment were confirmed by the evaluation of the sensorimotor status a) Using the forelimb use asymmetry test we found that lesioned rats were significantly impaired in the right forepaw use compared to controls (p = 0.025) and compared to the left paw (p = 0.015). In contrast, rats treated with hUCB cells showed no differences compared to controls (p = 0.981) and no differences in use between left and right paw (p = 0.184). Bars represent s.e.m. b) Recording footprints of the hindpaws revealed a reduced toespread indicative of spastic paresis in lesioned rats compared to controls (p =  0.038), but normal toespread not different from controls in hUCB-treated rats (p =  0.592). Bars represent s.e.m. Number of rats used: control group n =  10, lesion group n =  19, hUCB group n =  6.

The beneficial effects of the hUCB treatment, which resulted in a normalization of cortical processing, were further substantiated by the evaluation of the sensorimotor status of each individual rat (Fig. 6). Using the forelimb use asymmetry test [37] we quantified the percentage of impaired vs. non-impaired forelimb use during spontaneous vertical explorative behaviour. We found that the use of right forepaws in lesioned rats was significantly impaired as compared to controls (lesion: 7%±1%, control 14%±3%; p =  0.025). In contrast, rats treated with hUCB cells (transplantation group) showed no differences compared to controls (treated: 14%±4%, control 14%±3%; p =  0.981). In addition, footprints were recorded to assess sensorimotor skills of the hindpaws. Toe distance 1–5 was significantly reduced in lesioned rats (1.75±0.01 cm) compared to controls (1.87±0.02 cm), indicative of development of spastic paresis due to the lesion. In hUCB-treated rats, we observed normal toe distances (1.82±0.02 cm), which did not differ significantly from controls (p =  0.592).

## Discussion

Our data demonstrate that transplanted hUCB cells migrate from the intraperitoneal cavity to the hypoxic-ischemic lesion and settle down in the vicinity of damaged brain areas (Fig. 2). This is in line with previous findings in this and other animal models of brain injury [8], [9], [10], [12]. As shown by our data, the presence of hUCB cells in the damaged brain, which can be detected near the lesion as early as 24 hours after injection [38], seems to exert positive effects on the behavioural outcome after hypoxic-ischemic brain damage in neonatal rats. As neural differentiation of transplanted cells was not observed in this model (Meier et al., 2006; unpublished data), the effects observed in this study are likely to be mediated by an indirect action. We have now shown that the physiological correlate of sensorimotor improvement can be found in a reestablishment of cortical maps and RFs as well as paired-pulse behaviour. The presence of low-threshold cutaneous cortical responses argues for a significant preservation of the somatosensory pathway, even in animals where major changes in map and RF size, and paired-pulse behavior has been observed. However, further experiments using retrograde transported tracer to label somatosensory axons will provide anatomical evidence concerning the nature of afferent inputs.

Shrinkage and deterioration of cortical representations are momentous consequences typically observed after hypoxic brain damage, which have pathological consequences for sensorimotor outcome. After hUCB transplantation, cortical map size was largely restored almost matching that found in healthy animals (Fig. 3). As the structural damage after lesion was comparable between untreated and treated animals (Fig. 2), and both area of the lesioned hemisphere and lesion volume remained unaltered following transplantation [8], it is unlikely that a rescue of tissue is responsible for the map restoration after hUCB treatment. Instead, we assume that the cortical capacity for plasticity and recovery, which is restricted after lesion, is partially restored after hUCB treatment leading to a re-establishment of the map. The restriction of recovery of cortical function and structure after lesion is a well known challenge, which is, besides the general effects of cell death and inflammation, in part mediated by the up-regulation of repulsive extracellular matrix molecules and activation of astrocytes [39], [40]. In fact, transplantation of hUCB cells was shown to reduce the amount of pro-inflammatory cytokines in the lesioned brain [12]. Thus, it is feasible that hUCB cells affect inflammation and reactive gliosis by suppression of inflammatory proteins or secretion of anti-inflammatory factors as previously demonstrated in vitro [41]. In addition, recent in vivo studies provide evidence for an effect of hUCB cells on astroglial activation underlying glial scar formation (unpublished data) as well as on inflammation, apoptosis and neuroprotection [9] in the injured brain. These effects were also observed in response to the application of other sources of stem cells [42], [43]. Downstream, these mechanisms might synergistically provide the structural prerequisite allowing the injured map to recover.

Recently, following hUCB cell transplantation, rescue of striatal neurons has been reported after neonatal HI [44]. While it is conceivable that motor behaviour benefits from increased striatal neuron numbers, a contribution of the striatum to somatosensory processing appears less easy to explain. Moreover, the study had been performed at days 2 and 7 after ischemia, while our analysis was done at day 42. Most importantly, we found a significant number of transplanted hUCB cells in the vicinity of the insult in all lesioned/treated animals (Fig. 2).

Beside the hUCB cell mediated effects, an additional or alternative substrate subserving the remarkable sensorimotor recovery could be compensatory changes in the contralateral, intact hemisphere, or the emergence of afferent sensory inputs from the affected site shifting RFs into the ipsilateral hemisphere. In fact, both animal studies and studies in patients have provided convincing evidence for a role of the uninjured hemisphere in brain reorganization following brain injury or lesion [22], [45]. Our findings do not indicate any changes in the intact hemisphere. Thus, the observed cortical changes are likely to be due to a modulation in the injured hemisphere. However, as we tested 6 weeks after the insult, any changes that might have developed at an earlier phase or later in life would have gone undetected.

The decrease in cortical map size following lesion was accompanied by an increase in RF size, which was also restored in the transplantation group (Fig. 3). After hypoxic ischemic brain damage, a selective loss of GABAergic neurons has been reported [17], which is accompanied by an increase in extracellular glutamate [46]. Both effects lead to hyperexcitability in the damaged brain. The resulting deficits in GABAergic neuronal inhibition are in line with our observation of an increased RF size, as the size of cutaneous RFs is controlled by inhibitory neurons [47]. Similarly, increased glutamatergic and decreased GABAergic signalling might also result in the observed changes of paired pulse behaviour. While paired-pulse suppression was significantly decreased after hypoxic ischemic brain damage (Fig.5), hUCB transplantation led to the restoration of paired-pulse suppression at ISI_30_. The observation that hUCB treatment normalizes paired pulse-suppression at short, but not at longer ISIs might indicate that different mechanisms are involved in mediating the phenomenon of paired-pulse behavior at different ISIs. This view is compatible with a recent observation in aged rats, where no relationship between inhibition acting on RF size and on paired-pulse suppression has been found [33]; for an extended discussion of paired-pulse suppression see [33], [48]. Although the underlying mechanisms are still elusive, it is conceivable that transplantation of hUCB cells mediates the survival and rescue of GABAergic neurons and subsequent recovery of GABAergic inhibition via secretion of interleukins and growth factors. The capability of hUCB-derived mononuclear cells to produce substantial amounts of proteins including interleukins, chemokines, and growth factors has already been demonstrated in vitro [41], [49]. Interestingly, many of the secreted factors are known for their anti-inflammatory, anti-apoptotic, pro-angiogenic and neuroprotective effects. Thus, transplantation of hUCB cells might positively affect detrimental processes initiated by a hypoxic-ischemic brain lesion, possibly via secreted cytokines and associated with the modulation or reversal of lesion-induced electrophysiological parameters.

Map size and intactness of cortical representations are prerequisites for mediating adequate behavioural outcome [30], [31]. Therefore, given the remarkable impact of hUCB cells on a cell physiological level in SI, one could expect positive effects on sensorimotor behaviour. Indeed, our behavioural data showed a significant amelioration of the sensorimotor outcome in the same animals 7 weeks after the insult. This observation is in line with previously published data [8] and underlines again the positive effect of hUCB treatment for behavioural restitution.

Our data confirm a degradation of the somato-motor system following postnatal hypoxic-ischemic brain injury, and demonstrate for the first time beneficial effects following hUCB-treatment for somatosensory cortical processing. It appears conceivable that other systems are similarly affected. For example, auditory processing deficits have been described in rats with neonatal hypoxic-ischemic injury [50].

Our findings demonstrate that cortical processing and sensorimotor behaviour are largely normalized in response to hUCB cell transplantation after a hypoxic-ischemic brain lesion. Given the dependence of intact cortical processing on a maintained balance of excitatory and inhibitory mechanisms, our data suggest a beneficial role of hUCB treatment in their regulation, which might also be effective in other systems. Thus, by combining behavioural, histological and electrophysiological approaches, we here provide for the first time evidence for a link between cell transplantation and behavioural outcome via modulation of cortical reorganization and physiology.

## Materials and Methods

### Hypoxic-ischemic brain injury (HI)

The Levine model was used to achieve hypoxic-ischemic injury in neonatal rats, and was performed as described previously [8], [51]. Seven-day-old Wistar rat pups sustained a hypoxic-ischemic brain insult by ligation of the left common carotid artery, followed by an 80 min period of systemic hypoxia (8% oxygen, 92% nitrogen). In all experimental groups we used both male and female rats in about equal proportion. All surgical and experimental protocols have been approved by the appropriate institutional review committee (LANUV, Recklinghausen, Germany, 50.8735.1 109/4, 50.8735.1 109/5, and 50.10.32.08.048) and meet the guidelines of the German animal protection law. An overview about the time line of all experiments is given in Fig. 1.

#### Isolation of human umbilical cord blood-derived mononuclear cells

Blood from umbilical cord and placenta was obtained from the Depts. of Gynecology and Obstetrics (Ruhr-University Bochum, Elisabeth Hospital Bochum) after receiving the mother's written informed consent. The umbilical vein was punctured post partum, and the blood collected and stored as described previously [8], [51]. Preparation of the mononuclear cell fraction was performed by the Ficoll gradient technique (Amersham, Freiburg, Germany).

#### Experimental protocol

Randomly selected animals were assigned to three different experimental groups: control (no lesion), lesion (without transplantation), and transplantation group (lesion followed by transplantation of mononuclear cells). Animals of the lesion group (lesion only; n = 19) were subjected to ligation of the left common carotid artery, followed by systemic hypoxia. Twenty-four hours after the insult, this group received an intraperitoneal injection of 500 µl of 0.9% sodium chloride (sham injection). In the transplantation group (lesion followed by transplantation; n = 6), rats received 1×10^7^ human umbilical cord blood-derived mononuclear cells (in a volume of 500 µl 0.9% sodium chloride) by intraperitoneal injection twenty-four hours after the hypoxic-ischemic insult. The control group (no lesion; n = 10) comprised of animals that did not undergo any treatment or surgery. There was no use of immunosuppressants in any experimental group. Afterwards, animals were housed together with their littermates and mothers until postnatal day (P) 49.

#### Analysis of motor abilities

The day before the electrophysiological experiment at P48, behavioural analysis was performed. Footprint analysis was carried out in all animals as described previously [8], [37]. The glabrous skin of paws was colored, and footprints were printed onto paper when the rat walked along a defined gangway which was elevated by 30°. For each animal, 3 to 4 individual prints were measured regarding the distance between the first and fifth toe as described previously [8], [51]. A significant decrease in footprint width of the limbs contralateral to the lesion compared to that of the ipsilateral site was considered to reflect spastic paresis. Motor abilities of the forelimbs were also investigated by the cylinder-test, which determines forelimb use asymmetry. Rats were placed in a transparent cylinder (height 30 cm, diameter 19 cm), and videotaped for 3–10 minutes depending on the degree of movement. Analysis of the forelimb use was made using a video program (Adobe Premiere) with slow motion and frame-by-frame capabilities. The number of elevations of the right, left, or both forelimbs was counted.

#### Immunohistochemistry

Immunohistochemical analysis was performed as described previously [8]. Brains were dissected, covered in tissue freezing medium (Leica, Nussloch, Germany), and cryo-conserved in 8% methylcylcohexan in 2-methylbutan (v/v) (−80°C). Histology and immunohistochemistry were performed on cryosections of 12 µm thickness. Fluorescence was documented using conventional fluorescence microscopy (Zeiss 200 M inverted microscope including Apotome device). To determine the extent of brain injury, cryosections were stained with antibodies recognizing cleaved caspase 3 (1∶100; New England Biolabs, Frankfurt, Germany; indicative of neuronal cell death) and CD68 (ED1, 1∶100; Serotec, Düsseldorf, Germany; indicative of microglia activation). As hUCB cells are located within the area of activated astrocytes in vicinity of the lesion core [8], and as astrocytes are one source of chemoattractant SDF-1 [38], immunohistochemical detection of transplanted human cells was performed using HLA-DR antibodies (1∶50; Dako, Hamburg, Germany) in addition to the staining of rabbit-anti-human GFAP labeling astrocytes(1∶100; Sigma Aldrich, München, Germany).

#### Determination of the lesion volume

Lesion volume was calculated from the lesion area, determined in histologically stained sections of lesioned P49 rat brains with (n = 4) or without (n = 3) subsequent transplantation of hUCB using the ImageJ program (http://rsbweb.nih.gov/ij/index.html). The area of the lesion was calculated by subtracting the area of the lesioned hemisphere from the area of the non-lesioned hemisphere. Area measurements were performed in every 21^st^ section of the brain, starting with the most anterior section that showed characteristics of the brain lesion and finishing with the most posterior section affected by the lesion. Thus, the lesion volume was calculated from an average of 18.7±0.54 sections per animal, using the method according to Cavalieri [52]: LV = (n+1) x ST x ∑Ai with LV lesion volume, n number of sections between two planimetered sections, ST thickness of sections (12 µm), N number of planimetered sections, Ai lesion area.

#### Animal preparation and anaesthesia for electrophysiology

Experiments were performed in 35 adult Wistar rats of both sexes. Animals were anaesthetized with an initial dose of 0.0075 mg/g body weight Urethane (Sigma, 20% (w/v) in dist. H_2_O, i.p.), and were held under urethane anesthesia during the entire course of the experiment. Additional anaesthetic was administered when eye-blinking or paw-withdrawal reflexes could be elicited. Treatment of all animals was within the guidelines of the National Institutes of Health Guide and Care for Use of Laboratory Animals, all experiments were approved by the German Animal Care and Use Committee. The cisterna magna was drained to lower the intracranial pressure for minimizing pulsation and avoiding edema. After a bilateral craniotomy (0.5 cm rostral to 1 cm caudal from bregma) and resection of the dura, the cortex was covered with warm silicone oil (DC 200 50cst, Serva). Enlarged video images were taken from each hemisphere to use the blood vessels as landmarks for mapping. Rectal temperature was kept at 37°C using a feedback-controlled heating pad. The ECG and respiration were monitored throughout the experiments. Both hindlimbs were fixed to ensure a stable position for tactile stimulation of the glabrous side.

#### Electrophysiology

Glass micropipettes with a tip diameter of 10 µm (OD) filled with 3 M NaCl (1 MOhm at 10 kHz) and a low impedance reference electrode fixed to the neck muscles were used for recording. Electrode tip size was controlled by breaking tips to a defined size under microscopic control. Penetrations were made perpendicular to the cortical surface within and around the hindpaw representations of the primary somatosensory cortex using a motor micro drive (1 µm resolution) to advance the electrode. Cells or small clusters of cells were recorded extracellularly in layer IV of the hind paw representation of primary somatosensory cortex (SI) [22], [33] at approximately 700 µm subpial depth. Multiunit recordings consisted of 2 to 5 single units. Signals were amplified, high pass filtered and monitored on an oscilloscope and audio monitor. Action potentials were separated by amplitude discrimination and converted to TTL pulses, which were stored with a resolution of 500 µsec for further analysis together with TTLs controlling tactile stimulation on a PC running Spike 2 (Cambridge electronic design CED). Post-stimulus-time-histograms (PSTHs) could be displayed online. Penetrations were spaced at irregular intervals of 100–200 µm according to the vascular patterns, and penetration sites were marked in digitized pictures of the cortical surface in stereotactic coordinates. Borders of the hindpaw representation were defined by those cortical locations, where no reliable cutaneous response could be evoked, and were outlined in the cortical surface picture.

### Receptive field (RF) assessment and tactile stimulation

The size of cutaneous RFs on the glabrous skin of the hindpaw was measured by hand plotting [22], [33], [53] and by measuring length response profiles [53]. For hand plotting, RFs were defined as those areas on the glabrous skin at which just visible skin indentation (using small glass probes with a nodular tip of ∼2 mm diameter) evoked a reliable neuronal discharge [54]. Other studies have shown that just-visible indentation is in the range of 250 to 500 µm, which is in the middle of the dynamic range of cutaneous mechanoreceptors [55]. The location and the extent of RFs were transferred in a schematic drawing of the paw. Measures of RF size (area of skin in mm^2^) were obtained by planimetry. A total of 975 RFs were recorded (left hemisphere: control 127: lesioned97, treated 85; right hemisphere: control 192, lesioned 327, treated 147). Non-cutaneous (deep-input) RFs were infrequently encountered and were not included for further analyses.

To measure response length profiles [53], we recorded PSTHs after tactile stimulation using electromagnetic stimulators (Mini Shaker Typ 4810, Brüel&Kjær, Denmark) at three fixed positions (at the distal portion of a toe, at the pad, and at the proximal end of the heel, thus spanning the entire 35 mm of the paw axis). This analysis was restricted to RFs whose center was either at the very distal (on the toes) or proximal portion of the paw (on the heel). Stimulus duration was 5 ms delivered at 1 Hz, and neural responses were averaged over 64 repetitions. For obtaining a measure of RF length, neuronal responses (maximal peak response) were normalized according to the position evoking maximal response strength and plotted versus skin distance on the paw. Response length (in mm) was defined at that distance were responsiveness dropped to 50%. A total of 153 length profiles were recorded (left hemisphere: control 23: lesioned 24, treated 30; right hemisphere: control 24, lesioned 24, treated 28). Correlation analysis between RF size obtained by hand plotting and RF length revealed high correspondence (Pearson's linear correlation r = 0.77, p<0.0001, [53]) confirming the overall validity of determination of RF size by hand plotting.

### Paired-pulse behaviour

To measure paired pulse behaviour, pairs of tactile stimuli were applied to the center of a RF at interstimulus intervals (ISIs) of 30, 50, 80, 120 and 200 ms using the stimulation device described above. Renewal time was 2 sec. Each recording consisted of 64 trials. For further analysis, as measures of the paired-pulse behaviour, the ratios between the maximal response peaks (bin width 2 ms) of the second and first response peaks were calculated (A2/A1). Additionally we used the total number of spikes of the first and second response to calculate ratios between the integrals (I2/I1). A total of 386 paired pulse curves describing paired pulse behaviour at five different ISIs were recorded (left hemisphere: control 53: lesioned51, treated 41; right hemisphere: control 65, lesioned 129, treated 47).

#### Statistical analysis

All data were evaluated for normality and homogeneity of variances. Analysis was done by one-way ANOVA followed by parametric tests with Bonferroni correction where necessary. A probability of error less than 0.05 (p<0.05) was considered statistically significant. All data are given as mean +/− standard error of the mean (SEM) unless otherwise indicated.
